# Investigating the effect of emotional disclosure on the quality of life of women after abortion: a randomized controlled clinical trial

**DOI:** 10.1186/s40359-025-03355-y

**Published:** 2025-09-26

**Authors:** Mahla Salarfard, Maleknaz Ghannadkafi, Saeed Vaghee, Hamidreza Behnam Vashani, Kobra Mirzakhani

**Affiliations:** 1 Student Research Committee, Faculty of Nursing and Midwifery, Mashhad University of Medical Sciences, Mashhad, Iran; 2 Nursing and Midwifery Care Research Center, Mashhad University of Medical Sciences, Mashhad, Iran; 3 Department of Midwifery, School of Nursing and Midwifery, Mashhad University of Medical Sciences, Mashhad, Iran

**Keywords:** Disclosure, Emotion, Expressed, Quality of life, Abortion, Counseling

## Abstract

**Background:**

Women often experience various psychological complications following abortion, which significantly impair their quality of life. Emotional disclosure is one of the non-pharmacological methods for reducing psychological disorder symptoms. However, its specific effects in post-abortion contexts remain unclear. therefore this study aimed to evaluate the effect of emotional disclosure on quality of life among women after spontaneous abortion.

**Methods:**

In this randomized controlled clinical trial, 65 eligible women hospitalized due to spontaneous abortion at Valiasr and Shahid Rahimi hospitals in Birjand in 2021 were asked to complete demographic and quality-of-life questionnaires after providing informed consent. The women were randomly assigned to either the intervention or control group using a random sequence generated from the website (www.randomization.com*).* The control group received only the standard medical and education care of the hospital. The intervention group received the standard hospital education and medical care, along with the emotional disclosure intervention. After one individual training session, the intervention group performed four home writing sessions (15–20 min, twice weekly) with two weekly 15–20 min phone sessions with the researcher for verbal disclosure. The quality-of-life questionnaire was completed by both groups before, immediately after, and one month following the intervention. Data were analyzed using SPSS 22 statistical software, with a significance level set at *P* > 0.05. Statistical tests included the independent t-test, Mann-Whitney U test, repeated-measures analysis of variance (ANOVA), and chi-square test.

**Results:**

The results indicated that the intervention group demonstrated a significant improvement in the physical (*P* = 0.03), mental, and environmental (*P* = 0.01) domains of quality of life compared to the control group. However, no significant difference was observed in the social relationships aspect, either immediately after the intervention (*P* = 0.063) or one month later (*P* = 0.054), between the two groups.

**Conclusion:**

The study found that emotional disclosure significantly improved physical, psychological, and environmental quality of life domains in women after abortion, but had no significant effect on social relationships.

**Trial registration:**

This research project was registered at the Iranian Registry of Clinical Trials, IRCT registration number: IRCT20210528051421N1, Registration date: 2021-06-29.

**Supplementary Information:**

The online version contains supplementary material available at 10.1186/s40359-025-03355-y.

## Introduction

Abortion represents one of the most frequent complications of pregnancy, occurring in 11–31% of cases depending on population characteristics [1]. Defined as pregnancy termination before 20 weeks gestation or with fetal weight < 500 g, abortion carries significant physical and psychological consequences [2]. Recent studies indicate that over 50% of women experience emotional and psychological complications during the weeks and months following an abortion [3]. Women with recent spontaneous abortion are particularly vulnerable to negative outcomes, including pregnancy-related distress, sleep disorders, interpersonal sensitivity, psychosis, marital distress, suicide attempts, and substance abuse [4].

These physical and psychological sequelae profoundly affect quality of life (QoL) – a multidimensional construct encompassing physical, mental, and social health dimensions [5, 6] that serves as a critical healthcare indicator [7]. Notably, post-abortion women demonstrate poorer QoL across all domains (physical functioning, emotional well-being, and social relationships) compared to the general population [8, 9]. Given this significant QoL impairment, identifying effective psychological interventions becomes paramount. Among evidence-based approaches, emotional disclosure has emerged as a particularly promising method for QoL improvement [6].

Building upon this therapeutic potential, emotional disclosure – a well-established non-pharmacological intervention with roots in Aristotle’s concept of “psychological refinement” [10]. – addresses a key pathological mechanism: substantial evidence indicates that suppressing negative experiences contributes to both physical and mental health complications. This intervention involves expressing one’s deepest emotions either verbally (to others or oneself) or through writing [11, 12]. What makes this approach particularly valuable are its distinct advantages: teachability, self-administered format, absence of in-person visit requirements, and cost-effectiveness compared to conventional therapies [6].

The efficacy of emotional disclosure is supported by empirical evidence across populations. Akbari et al. (2015) demonstrated its effectiveness in enhancing QoL among cancer patients [6], while Kim et al. (2021) found it improved depression and fertility-related QoL in infertile patients [13]. Barzegar Amiri et al. (2018) reported significant reductions in depression, anxiety, and stress following open-heart surgery [14]. However, the literature presents some contradictions; Lepore et al. (2015) found no significant QoL improvement from written disclosure in colorectal cancer patients [15], highlighting the need for population-specific investigations.

Given these considerations – the high prevalence of spontaneous abortion, its profound health impacts, and the mixed evidence regarding emotional disclosure’s effectiveness – developing accessible interventions to improve post-abortion QoL represents a critical public health priority. This study therefore aimed to determine the effect of emotional disclosure on quality of life among women following spontaneous abortion.

## Method

This parallel-design randomized controlled clinical trial was conducted after obtaining ethical approval (IR.MUMS.NURSE.REC.1399.093) from Mashhad University of Medical Sciences and registration in the Iranian Registry of Clinical Trials (IRCT20210528051421N1; registration date: 2021-06-29). The study enrolled hospitalized post-abortion women at Wali Asr (AS) and Shahid Rahimi hospitals in Birjand between June and September 2021.

Participants were selected via convenience sampling from women admitted within 48 h post-abortion. We calculated the required sample size using the two-group mean comparison formula:

$$\:N=\frac{{\left({z}_{1-\alpha\:/2}+{z}_{1-\beta\:}\right)}^{2}({s}_{1}^{2}+{s}_{2}^{2})}{{\left({x}_{1}-{x}_{2}\right)}^{2}}$$

with 95% confidence level (z₁₋α/₂ = 1.96), and 80% power (z₁₋β = 0.84), with pilot study parameters (X₁ = 37.6, X₂ = 42.1, s₁ = 7.4, s₂ = 5.5). This yielded 32 participants per group. Accounting for 10% attrition, we enrolled 35 women per group.”

The inclusion criteria consisted of: willingness to participate in the study, being an Iranian national aged 18–45 years, having basic literacy, experiencing spontaneous abortion within 24–48 h prior to enrollment, having a wanted pregnancy, absence of chronic physical conditions (including cardiovascular diseases, diabetes, thyroid disorders, kidney diseases, or neurological conditions), no physical disabilities (such as hearing impairment, speech disorders, or inability to write), no history of mental health disorders (including depression, psychosis, bipolar disorder, or personality disorders), no exposure to major stressful events in the preceding six months, no use of sedatives or psychotropic medications during the last six months, no substance abuse (including drugs, tobacco, or alcohol), being married and cohabiting with one’s spouse, and having no history of infertility. Exclusion criteria included: experiencing significant stressful events during the study period, completing fewer than two written emotional disclosure sessions within two weeks, developing severe abortion-related complications requiring hospitalization during the study, failure to respond to researcher phone calls, voluntary withdrawal from the study, or becoming pregnant during the research period.

After obtaining approval for the research proposal and securing an official letter of introduction from the university’s vice-chancellor, the researcher attended Vali-Asr and Shahid Rahimi hospitals in Birjand. Upon presenting the university’s formal introduction letter and explaining the study’s objectives to the relevant authorities, the researcher was granted access to the gynecology and obstetrics wards.

Next, eligible participants were selected from women admitted to these wards within the past 48 h due to spontaneous abortion. The researcher introduced herself to potential participants, thoroughly explaining the study’s purpose and methodology. To encourage cooperation, participants were assured of the confidentiality of all written and verbal data. Following this, informed consent was obtained, and the research selection form was administered by the researcher.

An independent statistician (Statistician 1) with no other involvement in the trial generated the random allocation sequence using block randomization with varying block sizes equentially. numbered, opaque, sealed envelopes containing assignments were prepared and stored securely. For each enrolled participant, the same independent statistician opened the next envelope in sequence and communicated the assignment (Group A/B) to the study coordinator. Throughout this process, a separate statistical analyst (Statistician 2), who remained blinded to group allocations, conducted all data analyses using anonymized datasets where groups were coded as X and Y until completion of the final analysis. Participants and research investigator were not blinded to group assignment due to the nature of the intervention. However, the statistical analyst responsible for outcome assessment remained blinded to group allocations until final data analysis to minimize bias. Women who met the inclusion criteria were then asked to complete a demographic and midwifery information form and The World Health Organization Quality of Life questionnaire (short form).

The control group received only the standard medical care and education routinely provided to hospitalized women during their stay. This routine education included information on medication use, warning signs, follow-up procedures for receiving pathology results from laboratory tests, and the scheduled time for revisiting the physician after discharge. The intervention group received the standard hospital education and medical care, along with the emotional disclosure intervention. The emotional disclosure intervention involved written self-expression sessions where participants were instructed to write about their deepest emotions and thoughts regarding a significant negative emotional experience (e.g., pregnancy loss). participants could either focus on a single topic across sessions or address different themes each time (e.g., unresolved grief, relational impacts, or future anxieties). participants in the intervention group received a single individual training session on emotional disclosure techniques. This face-to-face session was conducted in the hospital by the researcher, who provided comprehensive instruction using an explanatory approach. Following the initial instruction, the intervention group was asked to complete four 15–20 min independent writing sessions over two consecutive weeks (two sessions per week) in a quiet, comfortable space at home, where they freely expressed their deepest negative emotions and feelings without secrecy on provided worksheets. Participants asked to submit dated written sheets after each session as physical proof of completion. They were encouraged to explore topics they frequently worry about, avoid, or that profoundly impact their lives, without concern for grammar, spelling, or external judgment. The researcher conducted brief weekly check-ins (via phone/email) to verify compliance, assess the quality and content of sessions, and address any issues. Quality and content were evaluated based on participants’ adherence to the training protocol, their ability to correctly implement the learned material, and their self-reported experiences. During these check-ins, the researcher evaluated whether the emotional disclosures were being performed as intended, both in terms of depth of emotional expression and adherence to protocol. At the end of each week, with prior coordination, the researcher conducted a 15–20 min verbal emotional disclosure session via telephone (two sessions total). During these calls, participants verbally expressed their emotions while the researcher actively listened. Additionally, during each session, the researcher monitored the proper implementation of the intervention protocol. In total, the intervention comprised 6 sessions: 4 written emotional disclosure sessions (2 per week) and 2 verbal emotional disclosure sessions (1 at the end of each week).

To evaluate the intervention’s sustained effects, the quality of life questionnaire was re-administered to both control and intervention groups immediately post-intervention and at one-month follow-up. Following completion of post-test assessments and in accordance with ethical principles, participants in the control group received the emotional disclosure protocol as an educational pamphlet.

The data collection tools in this study included the participant selection form, the demographic-obstetric information questionnaire, and the World Health Organization Quality of Life Questionnaire (WHOQOL). The participant selection form contained the study’s inclusion and exclusion criteria and consisted of 16 items, which were developed based on the research objectives, a review of relevant literature, and consultations with supervisors and advisors. the demographic and obstetric characteristics form included a comprehensive 18-item questionnaire. these forms were completed in paper format by the researcher through direct interviews in the maternity and gynecology wards of Shahid Rahimi and Valiasr hospitals in Birjand and medical record reviews.

To ensure content validity, each form was designed after reviewing the latest books and articles related to the research topic, under the supervision of academic advisors, and was then evaluated by seven expert faculty members from the Mashhad Nursing and Midwifery School. After incorporating their feedback and making necessary revisions, the final versions were approved for use. Since these forms contained clear, objective statements, their reliability was considered confirmed without requiring further statistical testing.

The World Health Organization Quality of Life Questionnaire - short form (WHOQOL-BREF) is a 26-item self-report questionnaire derived from the WHOQOL-100 instrument. It assesses four key dimensions: (1) Physical health (7 items: questions 3, 4, 10, 15, 16, 17, 18), (2) Psychological health (6 items: questions 5, 6, 7, 11, 19, 26), (3) Social relationships (3 items: questions 20, 21, 22), and (4) Environmental factors (8 items: questions 8, 9, 12, 13, 14, 23, 24, 25). The first two questions serve as general evaluators of overall quality of life and health status without belonging to any specific domain. All questions use a 1–5 Likert scale where lower scores indicate more problems and higher scores reflect better status, with consistent question composition across different cultures. The questionnaire’s construct validity was previously established by Nejat et al. (2006) [16], and in our study we confirmed its validity through content validation. Regarding reliability, Nejat et al. (2006) reported Cronbach’s alpha coefficients of 0.77 for physical health, 0.77 for psychological health, 0.75 for social relationships, and 0.84 for environmental factors [16]. In our present study, we similarly confirmed the questionnaire’s reliability with Cronbach’s alpha values exceeding 0.7 for all domains. The Quality of Life questionnaires were completed in paper format by the researcher, initially in the obstetrics and gynecology wards of the hospitals and in subsequent stages through scheduled appointments.

The data collected through questionnaires from both intervention and control groups were entered into the computer and analyzed using SPSS version 22 statistical software with a 95% confidence level. Descriptive statistics including frequency distribution tables, means, and standard deviations were used to characterize the research sample. The normal distribution of quantitative variables was assessed using the Kolmogorov-Smirnov test. Subsequent analyses were performed using independent t-tests, chi-square tests, Mann-Whitney tests, and repeated measures analysis of variance, with a *p*-value of less than 0.05 considered statistically significant.

## Results

**Fig. 1:**
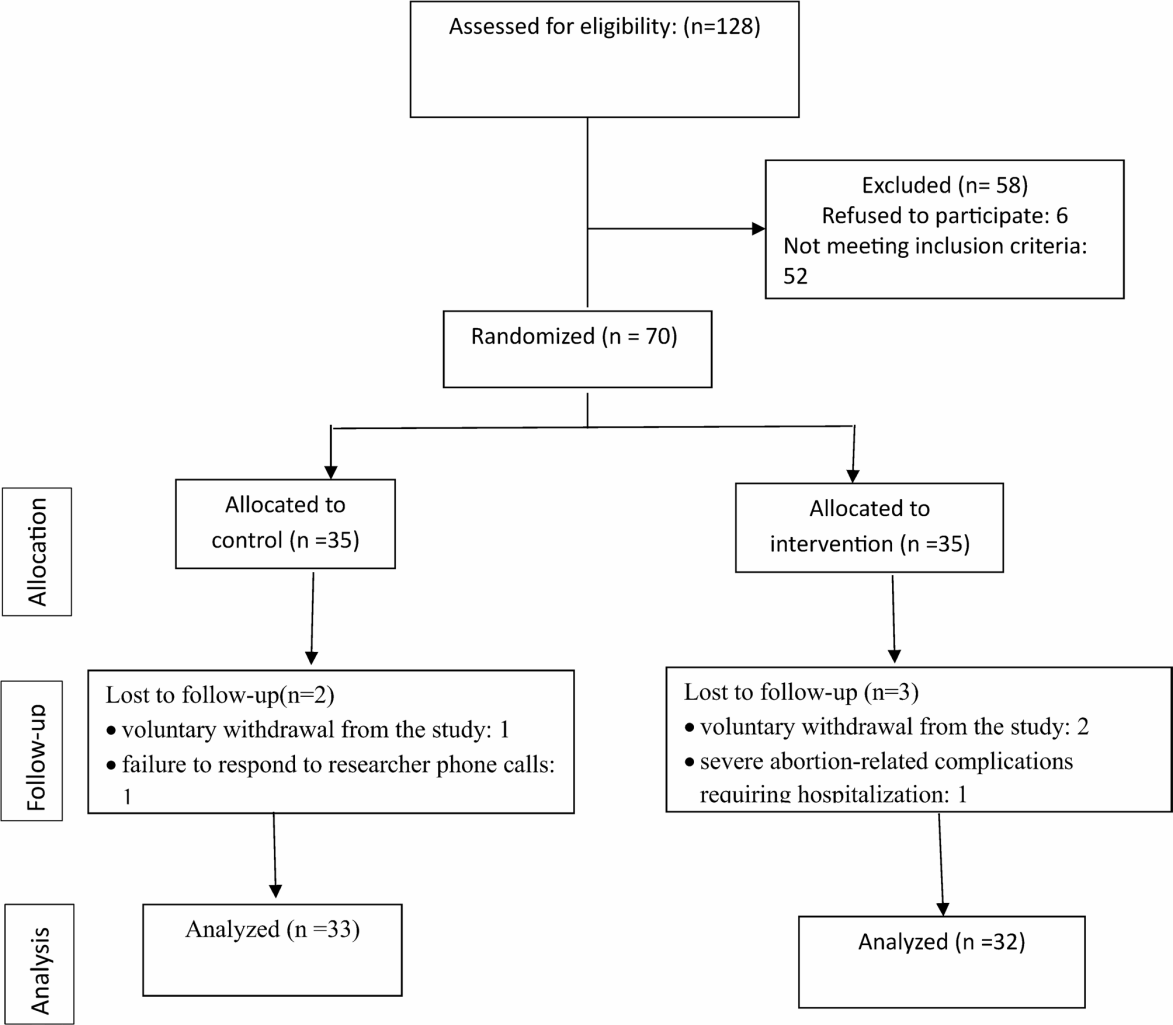
Study flow chart and sampling process

A flowchart of the participant sampling process is provided in Fig. 1. The age range of participants was 18 to 43 years, with mean ages of 31.4 ± 7.0 years in the intervention group and 32.4 ± 6.3 years in the control group, showing no significant difference (*P* = 0.500). Regarding education, most participants in both the intervention (53.1%) and control (51.5%) groups had education levels below high school diploma, with no significant between-group difference (*P* = 0.701). The majority of participants were housewives (78.1% intervention vs. 69.7% control, *P* = 0.513), and similar proportions in both groups reported sufficient monthly income (75.0% vs. 75.7%, *P* = 0.919). Additional analyses revealed no significant differences in spouse’s education level (*P* = 0.615), number of pregnancies (*P* = 0.790), number of deliveries (*P* = 0.452), history of abortion (*P* = 0.341), or duration of marriage (*P* = 0.611).

The independent t-test revealed no significant between-group difference in quality of life scores at baseline (*p* = 0.11). However, post-intervention assessments showed significantly higher quality of life scores in the intervention group compared to controls both immediately after the intervention (*p* = 0.007) and at the one-month follow-up (*p* = 0.006) (Table 1).

**Table 1:** Mean (± SD) quality of life scores among women post-abortion at baseline, immediately post-intervention, and one-month follow-up, by study group

Group Time	Intervention (32 women)		control (33 women)		The result of independent t test	
	Mean	SD	Mean	SD	t	*p*
Before intervention	90.1	11.8	89.5	8.5	2.6	0.11
Immediately after the intervention	97.9	11.5	90.1	11.1	2.80	0.007
One month after the intervention	99.3	11.7	91.3	11.0	2.82	0.006

In addition to the total quality of life score, we analyzed its four dimensions. For physical health, no significant between-group difference existed at baseline (*P* = 0.059). However, the intervention group showed significantly higher scores than controls both immediately post-intervention (*P* = 0.027) and at one-month follow-up (*P* = 0.038). Similarly, mental health scores revealed no baseline difference (*P* = 0.066) but demonstrated significant improvements in the intervention group at immediate (*P* = 0.031) and one-month (*P* = 0.023) assessments.

Regarding social relationships, we observed no significant differences at any time point (baseline: *P* = 0.077; immediate: *P* = 0.063; one-month: *P* = 0.054). For the living environment dimension, a significant baseline difference existed (*P* = 0.016), with the intervention group maintaining higher scores post-intervention (immediate: *P* = 0.019; one-month: *P* = 0.017) (Table 2).

Due to the non-homogeneity of pre-intervention living environment scores between groups, we employed analysis of covariance (ANCOVA) to control for baseline differences. The ANCOVA results demonstrated that even after adjusting for pre-intervention living environment scores as a covariate, statistically significant differences remained between the two groups in both the immediate post-intervention phase (*P* = 0.034) and at the one-month follow-up assessment (*P* = 0.042).

**Table 2:** Mean (± SD) scores of quality of life dimensions among post-abortion women at baseline, immediately post-intervention, and one-month follow-up, by study group

Quality of life dimensions	Time	Intervention (32 women)		control (33 women)		P
		Mean	SD	Mean	SD	
Physical health	Before intervention	25.90	4.30	23.87	4.20	0.059
	Immediately after the intervention	26.62	4.03	24.33	4.11	0.027
	One month after the intervention	27.21	3.78	25.18	3.94	0.038
Mental health	Before intervention	22.03	2.94	20.72	2.67	0.066
	Immediately after the intervention	22.15	2.79	20.69	2.54	0.031
	One month after the intervention	22.50	2.82	20.96	2.44	0.023
Social relationships	Before intervention	11.53	1.90	10.63	2.01	0.070
	Immediately after the intervention	11.59	1.86	10.66	2.07	0.063
	One month after the intervention	11.59	1.91	10.63	2.01	0.054
living environment	Before intervention	29.34	4.64	26.69	3.99	0.016
	Immediately after the intervention	29.34	4.65	26.75	3.97	0.019
	One month after the intervention	29.53	4.93	26.84	3.85	0.017

## Discussion

Our results demonstrate that emotional disclosure significantly enhanced post-abortion quality of life in three key domains: physical health, mental health, and living environment. However, no significant improvement was observed in social relationships, suggesting this dimension may require targeted interventions addressing external stigma or partner dynamics.

The significant improvements in physical and mental health following emotional disclosure align with inhibition theory. Women post-abortion often suppress emotions due to stigma, leading to chronic stress and physiological strain (e.g., elevated cortisol). Disclosure may have disrupted this cycle by releasing pent-up emotions, thereby reducing stress-related somatic symptoms (e.g., fatigue, pain) and enhancing psychological well-being. This mirrors findings in trauma populations, where written disclosure correlates with improved immune function and fewer physician visits [17]. Notably, our cohort—facing moralized stigma—may derive greater benefit from emotional release than groups with medically legitimized conditions (e.g., cancer), where stigma is less salient.

While our intervention successfully enhanced physical and mental health domains, its failure to improve social relationships may reflect a design limitation: unlike studies demonstrating social benefits (e.g., Akbari et al., 2015) [6], we employed individual rather than group-based disclosure. This distinction suggests that the therapeutic mechanisms operating in social QoL improvement may require communal validation - the very element absent in our protocol. When women processed emotions privately, they gained personal insight but lacked the restorative experience of having their narratives witnessed and normalized by peers facing similar stigma. This interpretation aligns with social identity theory, where group interventions combat isolation by fostering shared identity [18].

Consistent with our findings, both Akbari et al. (2015) and Yazdanfar et al. (2015) reported significant quality-of-life improvements following emotional disclosure interventions—though with domain-specific variations. Yazdanfar et al. (2015) designed a quasi-experimental study in which experimental group participants performed weekly 15–30 min written emotional disclosure exercises at home for one month, focusing on negative emotional experiences. Their results demonstrated significant improvements in quality of life measures compared to the control group [5]. Akbari et al. (2015) demonstrated in their quasi-experimental study of 30 married breast cancer patients (aged ≥ 38 years) that a combined oral/written emotional disclosure intervention (four 30-minute sessions) significantly improved physical, cognitive, social, and emotional functioning (*p* < 0.05), while showing no significant effects on sexual function or role performance [6]. their findings revealed the selective efficacy of emotional disclosure across quality-of-life domains. This pattern of domain-specific outcomes parallels our observations, suggesting that disclosure interventions may differentially impact various aspects of wellbeing. While Akbarī et al. (2015) found emotional disclosure improved social functioning in breast cancer patients, our study showed no significant social benefits for post-abortion women. An important methodological difference may explain the divergent social outcomes: Akbari et al.’s (2015) intervention was delivered in group sessions, while ours used individual disclosure. The group format likely provided built-in social reinforcement—participants practiced sharing and received immediate peer support. In contrast, our individual approach, while effective for personal processing, lacked this natural pathway to social reintegration, particularly for women facing abortion-related stigma.

Lepore et al. (2015) examined the effects of written emotional disclosure on 193 colorectal cancer patients. Participants were assigned to either an experimental group (writing about cancer-related emotions for 15 min twice weekly) or a control group (writing about neutral daily activities). Despite high adherence (81%) and participants reporting the writing as meaningful and personal, the study found no significant improvements in depression symptoms or overall quality of life measures post-intervention [15]. The authors concluded that written disclosure alone was ineffective as a standalone psychological intervention for this population, particularly for male participants and those who felt constrained in openly expressing cancer-related concerns. The discrepancy between our findings and those of Lepore et al. (2015) may reflect a critical methodological distinction: while their study relied exclusively on written disclosure, our intervention combined both written and verbal emotional expression. Written disclosure alone—though valuable for private reflection—lacks the interpersonal validation and real-time cognitive reframing that verbal sharing provides. For women coping with post-abortion stigma, our two-stage approach - initial emotional organization through writing followed by affirmation through verbal sharing - created a more robust therapeutic process than written disclosure alone. The writing phase helped structure chaotic thoughts and feelings, while the verbal component provided essential social validation that reduced isolation and self-blame. The gender composition differences between studies offer another plausible explanation for the divergent outcomes. While our study exclusively examined women - who demonstrate greater emotional disclosure tendencies and typically benefit more from expressive interventions (Kowalski, 1999) [19]- Lepore et al.‘s (2015) sample comprised equal numbers of men and women (approximately 50% male). This is particularly significant given established gender differences in emotional processing: men often resist self-disclosure due to masculine norms emphasizing self-reliance, especially in medical contexts where vulnerability may be stigmatized. Our all-female cohort, by contrast, represented a population both more likely to engage authentically with disclosure and more culturally permitted to seek emotional support - factors that may have potentiated our intervention’s effects [20] .

This research has limitations. First, the data were collected through self-report measures, meaning the accuracy of responses relied on participants’ honesty. This method introduces potential response biases, which objective measures could have mitigated. To mitigate this, we emphasized the confidentiality of their information to encourage truthful answers. Second, the study was conducted with a relatively modest sample size drawn from only two hospital settings, which may affect the generalizability of the results to broader populations. third, while the intervention showed promising effects during the one-month follow-up period, the absence of longer-term assessment leaves its sustained impact uncertain. Additionally, It was not possible to implement blinding in this study, leaving open the possibility of expectation effects influencing outcomes.

## Conclusion

The findings indicate that emotional disclosure had a significant positive effect on certain domains of quality of life, including physical, psychological, and environmental aspects, among women after abortion. However, no significant improvement was observed in the social relationships domain, as evidenced by the lack of statistically meaningful differences between the intervention and control groups in both post-intervention and follow-up assessments. These results suggest that implementing emotional disclosure as a routine component of post-abortion care could substantially enhance women’s wellbeing, though should be combined with targeted social support interventions to address relational challenges stemming from persistent stigma. Therefore, it is recommended that similar interventions, including emotional disclosure conducted in group settings, be carried out over a longer period of time and in diverse geographical and cultural contexts in different regions of the country.

## Supplementary Information

Below is the link to the electronic supplementary material.

Supplementary Material 1

Supplementary Material 2

## Data Availability

No datasets were generated or analysed during the current study.

## References

[1] Cunningham FG, Leveno KJ, Dashe JS, Hoffman BL, Spong CY, Casey BM, et al. Williams obstetrics. 26nd ed. New York: McGraw-Hill Education; 2022.

[2] Wijesooriya LRA, Palihawadana T, Rajapaksha R. A study of psychological impact on women undergoing miscarriage at a Sri Lankan hospital setting. Sri Lanka J Obstet Gynecol. 2015;37(2).

[3] Tulandi T, Al-Fozan HM. Spontaneous abortion: Risk factors, etiology, clinical manifestations, and diagnostic evaluation. UpToDate. 2011.

[4] Balducci C, Avanzi L, Fraccaroli F. Emotional demands as a risk factor for mental distress among nurses. La Medicina Del Lavoro. 2014;105(2):100–8. 24909042

[5] Yazdanfar M, Manshaee G, Agah Herris M, Alipour A, Noorbala A. The effectiveness of written emotional disclosure training on psychological well-being and quality of life in psychosomatic disorders. J Res Health. 2015;5(1):35–41.

[6] Akbari Nakhjovani H, Badri Gargari R. Efficacy of methods of emotional disclosure (verbal/written) to improve the quality of life in women with breast cancer. Stud Med Sci. 2015;26(6):519–30.

[7] Kuehner C, Buerger C. Determinants of subjective quality of life in depressed patients: the role of self-esteem, response styles, and social support. J Affect Disord. 2005;86(2–3):205–13. 15935240 10.1016/j.jad.2005.01.014

[8] Carter J, Applegarth L, Josephs L, Grill E, Baser RE, Rosenwaks Z. A cross-sectional cohort study of infertile women awaiting oocyte donation: the emotional, sexual, and quality-of-life impact. Fertility and Sterility. 2011;95(2):711-6.e1. 10.1016/j.fertnstert.2010.10.004 21055740

[9] Tavoli Z, Mohammadi M, Tavoli A, Moini A, Effatpanah M, Khedmat L, et al. Quality of life and psychological distress in women with recurrent miscarriage: a comparative study. Health Qual Life Outcomes. 2018;16(1):150. 30055644 10.1186/s12955-018-0982-z PMC6064101

[10] izanlou M, Youseframaki M, shamsalinia A. Written emotional disclosure and its effect on health(A review article). J Casp Health Aging. 2023;8(2):0.

[11] Ahmadi Tahoor Soltani M, Ramezani V, Abdollahi MH, Najafi M, Rabiei M. The effectiveness of emotional disclosure (Written and Verbal) on symptoms of depression, anxiety and stress in students. J Clin Psychol. 2010;2(4):51–9.

[12] yousefi afrashteh m, Ayar F. The effect of written emotional disclosure on mindfulness and cognitive emotion regulation strategies. Res Cogn Behav Sci. 2023;13(1):23–42.

[13] Kim M, Hong J-E, Ban M. Mediating effects of emotional Self-Disclosure on the relationship between depression and quality of life for women undergoing In-Vitro fertilization. Int J Environ Res Public Health. 2021;18(12):6247. 34207782 10.3390/ijerph18126247 PMC8296028

[14] Amiri ZB, Sanagoo A, Jouybari L, Bahnampour N, Kavosi A. The effect of written emotional disclosure on depression, anxiety, and stress of patients after open heart surgery. BRAIN Broad Res Artif Intell Neurosci. 2019;10(2):55–64.

[15] Lepore SJ, Revenson TA, Roberts KJ, Pranikoff JR, Davey A. Randomised controlled trial of expressive writing and quality of life in men and women treated for colon or rectal cancer. Psychol Health. 2015;30(3):284–300. 25271396 10.1080/08870446.2014.971798 PMC4289438

[16] Nejat S, Montazeri A, Holakouie Naieni K, Mohammad K, Majdzadeh S. The world health organization quality of life (WHOQOL-BREF) questionnaire: translation and validation study of the Iranian version. J School Public Health Inst Public Health Res. 2006;4(4):1–12.

[17] Pennebaker JW, Smyth JM. Opening up by writing it down: how expressive writing improves health and eases emotional pain. Guilford; 2016.

[18] Khadka C. Social identity theory and group behavior. TUTA J. 2024:105–20.

[19] Kowalski RM. Speaking the unspeakable: Self-disclosure and mental health. 1999.

[20] Ranjbar BS, Karami J, Zabet M, Zalipour S. A B. Effectiveness of written emotional disclosure in the symptoms of postpartum depression in Iranian Mothers[text in Persian]. 2017.

