# Supplementary material for: Investigating the effect of emotional disclosure on the quality of life of women after abortion: a randomized controlled clinical trial
Source: BMC Psychol. 2025 Sep 26;13:1051. doi: 10.1186/s40359-025-03355-y (PMC12465752; doi:10.1186/s40359-025-03355-y)
Supplement: Supplementary file 2 — Supplementary Material 2 [file 40359_2025_3355_MOESM2_ESM.doc]

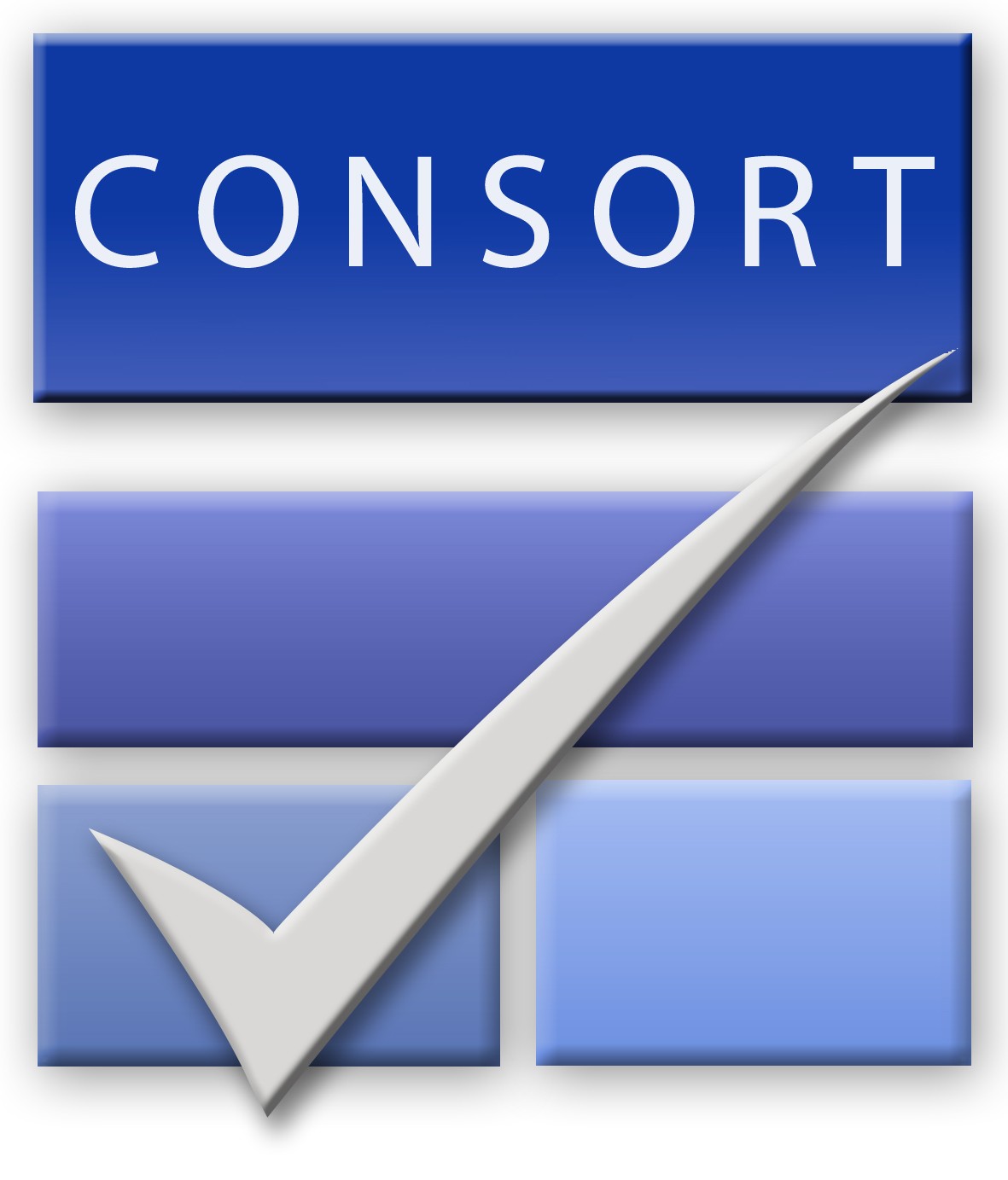
CONSORT 2010 checklist of information to include when reporting a randomised trial*

| Section/Topic | Item No | Checklist item | Reported on page No |
| --- | --- | --- | --- |
| Title and abstract | | | |
|  | 1a | Identification as a randomised trial in the title | Page 1, line 2 |
|  | 1b | Structured summary of trial design, methods, results, and conclusions (for specific guidance see CONSORT for abstracts) | Page 1-2, line 16-44 |
| Introduction | | | |
| Background and objectives | 2a | Scientific background and explanation of rationale | Page 2-3, line 70-81 |
|  | 2b | Specific objectives or hypotheses | Page 3, line 80-81 |
| Methods | | | |
| Trial design | 3a | Description of trial design (such as parallel, factorial) including allocation ratio | Page 3, line 83 |
|  | 3b | Important changes to methods after trial commencement (such as eligibility criteria), with reasons | No changes |
| Participants | 4a | Eligibility criteria for participants | Page 3, line 95-108 |
|  | 4b | Settings and locations where the data were collected | Page 3, line 86-87 |
| Interventions | 5 | The interventions for each group with sufficient details to allow replication, including how and when they were actually administered | Page 4, line 133-162 |
| Outcomes | 6a | Completely defined pre-specified primary and secondary outcome measures, including how and when they were assessed | Page 4, line 130-132 and Page 5, line 163-167 |
|  | 6b | Any changes to trial outcomes after the trial commenced, with reasons | No changes |
| Sample size | 7a | How sample size was determined | Page 3, line 88-93 |
|  | 7b | When applicable, explanation of any interim analyses and stopping guidelines | No interim analyses and stopping guidelines |
| Randomisation: |  |  |  |
| Sequence generation | 8a | Method used to generate the random allocation sequence | Page 4, line 120-122 |
|  | 8b | Type of randomisation; details of any restriction (such as blocking and block size) | Page 4, line 120-122 |
| Allocation concealment mechanism | 9 | Mechanism used to implement the random allocation sequence (such as sequentially numbered containers), describing any steps taken to conceal the sequence until interventions were assigned | Page 4, line 122-132 |
| Implementation | 10 | Who generated the random allocation sequence, who enrolled participants, and who assigned participants to interventions | Page 4, line 120-124 |
| Blinding | 11a | If done, who was blinded after assignment to interventions (for example, participants, care providers, those assessing outcomes) and how | Page 4, line 124--132 |
|  | 11b | If relevant, description of the similarity of interventions | Page 4, line 133-137 |
| Statistical methods | 12a | Statistical methods used to compare groups for primary and secondary outcomes | Page 6, line 199-205 |
|  | 12b | Methods for additional analyses, such as subgroup analyses and adjusted analyses | Not applicable |
| Results | | | |
| Participant flow (a diagram is strongly recommended) | 13a | For each group, the numbers of participants who were randomly assigned, received intended treatment, and were analysed for the primary outcome | Page 6, Fig 1 |
|  | 13b | For each group, losses and exclusions after randomisation, together with reasons | Page 6, Fig 1 |
| Recruitment | 14a | Dates defining the periods of recruitment and follow-up | Page 3, line 87 and Page 7, Table 1 |
|  | 14b | Why the trial ended or was stopped | Not stopped |
| Baseline data | 15 | A table showing baseline demographic and clinical characteristics for each group | Page 7, line 227-235 |
| Numbers analysed | 16 | For each group, number of participants (denominator) included in each analysis and whether the analysis was by original assigned groups | Page 6, Fig 1 |
| Outcomes and estimation | 17a | For each primary and secondary outcome, results for each group, and the estimated effect size and its precision (such as 95% confidence interval) | Page 7-8, Table 1 and 2 |
|  | 17b | For binary outcomes, presentation of both absolute and relative effect sizes is recommended | Not applicable |
| Ancillary analyses | 18 | Results of any other analyses performed, including subgroup analyses and adjusted analyses, distinguishing pre-specified from exploratory | Not applicable |
| Harms | 19 | All important harms or unintended effects in each group (for specific guidance see CONSORT for harms) | Not applicable |
| Discussion | | | |
| Limitations | 20 | Trial limitations, addressing sources of potential bias, imprecision, and, if relevant, multiplicity of analyses | Page 10, 332-340 |
| Generalisability | 21 | Generalisability (external validity, applicability) of the trial findings | Page 10, 341-352 |
| Interpretation | 22 | Interpretation consistent with results, balancing benefits and harms, and considering other relevant evidence | Page 10, 341-352 |
| Other information | | |  |
| Registration | 23 | Registration number and name of trial registry | Page 3, line 83-86 |
| Protocol | 24 | Where the full trial protocol can be accessed, if available | Not published |
| Funding | 25 | Sources of funding and other support (such as supply of drugs), role of funders | Page 11, line 365-366 |

Citation: Schulz KF, Altman DG, Moher D, for the CONSORT Group. CONSORT 2010 Statement: updated guidelines for reporting parallel group randomised trials. BMC Medicine. 2010;8:18.
© 2010 Schulz et al. This is an Open Access article distributed under the terms of the Creative Commons Attribution License (<http://creativecommons.org/licenses/by/2.0>), which permits unrestricted use, distribution, and reproduction in any medium, provided the original work is properly cited.

*We strongly recommend reading this statement in conjunction with the CONSORT 2010 Explanation and Elaboration for important clarifications on all the items. If relevant, we also recommend reading CONSORT extensions for cluster randomised trials, non-inferiority and equivalence trials, non-pharmacological treatments, herbal interventions, and pragmatic trials. Additional extensions are forthcoming: for those and for up-to-date references relevant to this checklist, see [www.consort-statement.org](http://www.consort-statement.org/).
